# Review article: the role of oxidative stress in pathogenesis and treatment of inflammatory bowel diseases

**DOI:** 10.1007/s00210-014-0985-1

**Published:** 2014-05-06

**Authors:** Aleksandra Piechota-Polanczyk, Jakub Fichna

**Affiliations:** Department of Biochemistry, Faculty of Medicine, Medical University of Lodz, Mazowiecka 6/8, 92-215 Lodz, Poland

**Keywords:** Free radicals, Crohn’s disease, Ulcerative colitis, Antioxidants, Corticosteroids, Anti-TNF-α antibodies

## Abstract

In this review, we focus on the role of oxidative stress in the aetiology of inflammatory bowel diseases (IBD) and colitis-associated colorectal cancer and discuss free radicals and free radical-stimulated pathways as pharmacological targets for anti-IBD drugs. We also suggest novel anti-oxidative agents, which may become effective and less-toxic alternatives in IBD and colitis-associated colorectal cancer treatment. A Medline search was performed to identify relevant bibliography using search terms including: ‘free radicals,’ ‘antioxidants,’ ‘oxidative stress,’ ‘colon cancer,’ ‘ulcerative colitis,’ ‘Crohn’s disease,’ ‘inflammatory bowel disease.’ Several therapeutics commonly used in IBD treatment, among which are immunosuppressants, corticosteroids and anti-TNF-α antibodies, could also affect the IBD progression by interfering with cellular oxidative stress and cytokine production. Experimental data shows that these drugs may effectively scavenge free radicals, increase anti-oxidative capacity of cells, influence multiple signalling pathways, e.g. MAPK and NF-kB, and inhibit pro-oxidative enzyme and cytokine concentration. However, their anti-oxidative and anti-inflammatory effectiveness still needs further investigation. A highly specific antioxidative activity may be important for the clinical treatment and relapse of IBD. In the future, a combination of currently used pharmaceutics, together with natural and synthetic anti-oxidative compounds, like lipoic acid or curcumine, could be taken into account in the design of novel anti-IBD therapies.

## Introduction

Cells are continuously threatened by the damage caused by reactive oxygen/nitrogen species (ROS/RNS), which are produced during physiological oxygen metabolism. Both ROS and RNS at low and moderate concentrations are signalling molecules involved in mitogenic response or in defence against infectious agents. However, excessive production of ROS and RNS or their inefficient scavenging leads to oxidative and nitrosative stress, respectively. This condition is potentially dangerous as it may alter inflammatory response and lead to lipid and protein modifications, DNA damage, apoptosis or cancerogenic cell transformation (Valko et al. 2001; Ridnour et al. 2005; Valko et al. 2007). Because of this, oxidative stress has been implicated in a number of human diseases, including inflammatory bowel diseases (IBD) and colorectal cancer.

This review will summarise the latest reports on the role of oxidative stress and oxidative stress-induced signalling pathways in the aetiology of ulcerative colitis (UC), Crohn’s disease (CD) and colitis-associated colorectal cancer. We will also focus on the effects of well-established therapeutics on oxidative stress and suggest future strategies for the treatment of free radicals production in UC, CD and colitis-associated colorectal cancer.

## Types and sources of free radicals in intestinal tissue

### Reactive oxygen species

**Fig. 1:**
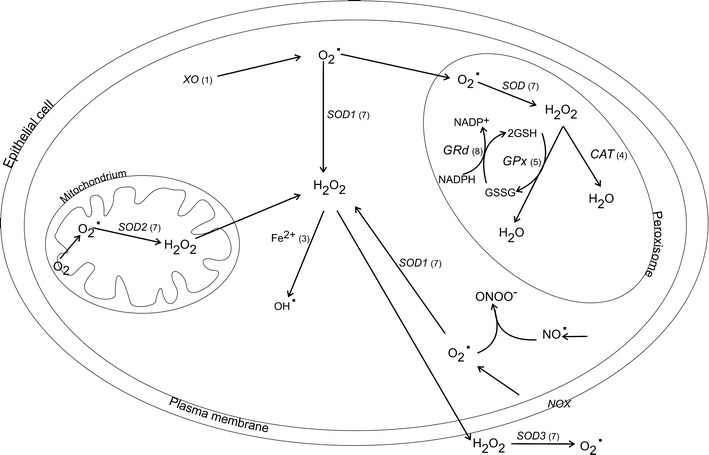
Formation of ROS and anti-oxidant defence system in intestinal epithelial cells. *CAT* catalase, *GRd* glutathione reductase, *GSH* reduced glutathione, *GSSG* oxidised glutathione, *GPx* glutathione peroxidise, *H*_*2*_ *O*_*2*_ hydrogen peroxide, *NO* ^•^ nitric oxide, *NOX* NADPH oxidase, *ONOO* ^*−*^ peroxynitrate, *O*_*2*_^*•−*^ superoxide anion, *OH* ^*•*^ hydroxyl radical, *SOD1* cooper/zinc superoxide dismutase, *SOD2* mitochondrial superoxide dismutase, *SOD3* extracellular superoxide dismutase, *XO* xanthine oxidase. *Numbers* corresponds to reactions catalysed by representative enzymes and presented in Table 2

The most abundant free radical in human tissues is the superoxide anion (O_2_•^−^), generated by the addition of one electron to molecular oxygen (Miller et al. 1990). Its main source in a cell is complex I and III of the mitochondrial electron transport chain, which converts 1–3 % of total oxygen to the superoxide anion (Muller et al. 2004) (Table 1 and Fig. 1 reaction (7)). Another source of O_2_•^−^ is an enzymatic reaction catalysed by xanthine oxidase (XO) [Fig. 1 reaction (1)] and membrane enzyme complexes named NADPH oxidases (NOX) (see Fig. 1). The NOX family comprises five isoforms, from which NOX1 is highly expressed in colon epithelium (Dutta and Rittinger 2010). When activated, NOX1 catalyses the transmembrane electron transport to two molecular oxygens forming O_2_•^−^. NOX1-induced O_2_•^−^ at the luminal surface of the colon has been suggested to enhance host defence (Geiszt et al. 2003). Moreover, NOX1 and NOX4 have been implicated as persistent, endogenous ROS generators that may contribute to the hepatitis C virus-related pathologies (de Mochel et al. 2010).

**Table 1:** Enzymatic reactions that participate in ROS/NOS generation in the GI tract

Enzyme	Reaction	Site of action	Reaction No. in Fig. 1
complex I and III/ubiquinone of the mitochondrial electron transport chain	Complex I (NADH dehydrogenase): O_2_ + NADH → O_2_^•−^ + NAD^+^ Complex III (cytochrome bc_1_): O_2_ → O_2_^•−^	Mitochondria	
Xanthine oxidase	Xanthine + O_2_ + NADPH → O_2_^•−^ + H_2_O_2_ + NADP^+^ + uric acid	Plasma and cytoplasm of epithelial cells	(1)
NADPH oxidase	2O_2_ + NADPH → 2O_2_^•^ + NADP^+^ + H^+^	Cell membrane	(2)
Haber-Weiss reaction	H_2_O_2_ + O_2_^•−^ → O_2_ + OH + OH^•^	Plasma and cell’s cytoplasm	
Fenton reaction	H_2_O_2_ + Fe^2+^ → Fe^3+^ + OH + OH^•^	Plasma and cell’s cytoplasm	(3)
Catalase (CAT)	2H_2_O_2_ → O_2_+ H_2_O	the cytoplasm and peroxisomes of epithelium and lamina propria; leukocytes.	(4)
Glutathione peroxidase (GPx)	H_2_O_2_ + 2GSH → GSSG + 2H_2_O	GPx1- peroxisomes of colon lymphatic tissue and the lamina propria, submucosa, muscularis and serosa;	(5)
		GPx2- peroxisomes of the luminal epithelium;	
		GPx3- secreted by the intestinal epithelial cells;	
		GPx4- peroxisomes of colonic and ileal tissues.	
Endothelial nitric oxide synthase (eNOS)	l-arginine + O_2_ → l-citrulline + NO^•^	Cell membrane of the endothelial cells	(6)
Inducible nitric oxide synthase (iNOS)	NO^•^ + O_2_^•−^ → ONOO^−^	Cytoplasm of inflammatory and epithelial cells	
Superoxide dismutase (SOD)	2H^+^ + 2O_2_^•−^ → O_2_ + H_2_O_2_	SOD1- cytoplasm and small amount in nucleus; SOD2- mitochondria; SOD3- plasma.	(7)
Glutathione reductase (GRd)	GSSG + NADPH → GSH + NADP^+^	Like GPx	(8)

Under stress conditions, O_2_^•−^ concentrations rise leading to excessive production of deleterious hydroxyl radical (OH^•^) through the Haber-Weiss reaction. The hydroxyl radical is also generated from hydrogen peroxide (H_2_O_2_) in the reaction catalysed by ferrous ion (Fe^2+^) [the Fenton reaction; Fig. 1 reaction (3)]. Instead of ferrous, other transient metals like copper, chromium or cobalt may participate in OH^•^ generation, those reactions become a significant source of OH^•^ under oxidative stress conditions or when the concentration of free, unbounded transient ions increases, e.g. during hemodialysis. In the gastrointestinal (GI) tract, OH^•^ inactivates a crucial mitochondrial enzyme pyruvate dehydrogenase, depolymerises GI mucin and inflicts mitochondrial RNA and DNA damages (Tabatabaie et al. 1996; Takeuchi et al. 1996; Halliwell 1999).

Another protonated form of O_2_^•−^ is perhydroxyl radical (HOO^•^), which initiates fatty acid peroxidation. Lipid peroxidation disturbs integrity, fluidity and permeability of biomembranes, modifies lipoproteins to pro-inflammatory forms and generates potentially toxic products. Moreover, lipid peroxidation products have been shown to possess mutagenic and carcinogenic properties (Poli et al. 2008; Winczura et al. 2012).

Apart from mitochondria, another source of free radicals in cells is plasma membrane NADPH oxidases or peroxisomes, which consume oxygen and produce H_2_O_2_. Under physiological conditions, peroxisome-derived H_2_O_2_ is converted to water by catalase (CAT) [Fig. 1 reaction (4)]. However, damaged peroxisome releases H_2_O_2_ directly to cytoplasm, therefore contributing to oxidative stress. Moreover, together with O_2_^•−^, H_2_O_2_ may be converted to highly toxic and oxidising OH^•^ hydrogen peroxide in Fenton and Haber-Weiss reactions (Fransen et al. 2012).

In the GI tract, O_2_^•−^ is mainly generated by XO [Fig. 1 reaction (1)]. It is consequently converted to H_2_O_2_ in the reaction catalysed by CAT and/or glutathione peroxidase (GPx) [Fig. 1 reaction (4) and (5), respectively]. H_2_O_2_ produced by neutrophils is subsequently utilised by myeloperoxidase (MPO) to produce hypochlorite ion (OCl^−^). Superoxide anion is a highly reactive, highly unstable, very short lived form of ROS which causes it to react away very quickly and makes it membrane impermeable; therefore, it acts near the place of its origin causing oxidation of surrounding biomolecules, while H_2_O_2_ can freely diffuse across cell membranes and oxidise compounds located further, e.g. membrane lipids of pathogens. The H_2_O_2_ diffusion in GI is facilitated by aquaporin 8 (Te Velde et al. 2008). Interestingly, basal level of ROS in enterocytes differs, with lower concentration of ROS in small intestine and higher in colon (Sanders et al. 2004). The differences in ROS generation may influence the levels of oxidised proteins, lipids and DNA damage, thus contributing to the higher susceptibility of colon to GI diseases at these two intestinal sites.

During pathological states, circulating XO binds to vascular endothelial cells and produces site-specific oxidative injury of the intestine tissue (Tan et al. 1993). Moreover, activated neutrophils undergo series of reactions termed “the respiratory burst,” in which O_2_^•−^ is generated. It was shown that this process incorporates NOX enzymes, especially NOX2, because NOX2 knockout mice have reduced oxidative burst and are less susceptible to experimentally induced ulcerative colitis (Bao et al. 2011).

### Reactive nitrogen species

The second group of free radicals are reactive nitrogen species that are by-products of nitric oxide synthases (NOS), which are expressed in selected cells of the intestinal submucosa and mucosal regions. NOS metabolises arginine to citrulline and forms the nitric oxide radical (NO^•^) via a five-electron oxidative reaction (Ghafourifar and Cadenas 2005). The nitric oxide radical has a relatively long half-life, but slow reaction time due to its rapid diffusion into the bloodstream and inactivation by haemoglobin. The nitric oxide radical is vital for proper functioning of an organism, as its physiological action includes neurotransmission, blood pressure regulation and immunomodulation. Furthermore, the vasodilatory actions of NO^•^ play a prominent role in the capillary recruitment of absorptive hyperaemia, catalysed by the endothelial NOS (eNOS) isoform, localised to the microvasculature at the submucosa–mucosa interface (Matheson et al. 2000). In addition, the nitric oxide radical protects epithelial cells against H_2_O_2_-induced toxicity and diminishes leukocyte adhesion to endothelial cells (Kim and Kim 1998; Binion et al. 2000). While eNOS produces NO^•^ in a pulsative way, the other NOS isoform termed inducible NOS (iNOS) produces NO^•^ in a constant manner. iNOS is detected only in inflamed tissue and is responsible for an excessive generation of RNS in activated macrophages, leukocytes and epithelial cells in the intestinal mucosa (Dijkstra et al. 1998). It was demonstrated that in UC, the activation of iNOS/cyclooxygenase-2 (COX-2)/5-lipooxygenase (5-LOX) loop and increased contents of their end products, namely NO, prostaglandin E_2_ (PGE_2_) and leukotriene B_4_ (LTB_4_), contribute to a damage of large intestine mucous membrane by overproduction of free radicals and impairment of anti-oxidative system (Sklyarov et al. 2011). Moreover, iNOS-derived NO reacts with tyrosine leading to nitrotyrosine production. It was indicated that patients with UC, but not collagenous colitis, have intense epithelial staining for nitrotyrosine associated with infiltration of neutrophils in the epithelium (Perner et al. 2001).

The reaction between NO^•^ with O_2_^•−^ leads to peroxynitrite production (ONOO^−^), which is an aggressive oxidising agent that can cause DNA fragmentation and lipid oxidation. Peroxynitrite is generated in cells containing NOS enzymes, such as smooth muscle or endothelial cells and, in particular during inflammatory response, by stimulated leukocytes.

## Lipid peroxidation and lipid radicals

Both ROS and RNS can contribute to lipid peroxidation. Particularly susceptible to oxidative damages are membrane lipids and lipoproteins since they are rich in polyunsaturated fatty acids. During lipid peroxidation, a hydroperoxy group is introduced into the hydrophobic tails of unsaturated fatty acids. This change can result in structural alterations of biomembranes and lipoproteins via disturbance of hydrophobic lipid-lipid and lipid-protein interactions, or can lead to generation of hydroperoxyl radicals and reactive aldehyde derivates, which may induce secondary modifications of other cell components. The end products of lipid peroxidation, like malondialdehyde or 4-hydroxynonenal, can cause protein damage by reactions with lysine amino groups, histidine imidazole groups or cysteine sulphydryl groups [see review (Catala 2009)].

Lipid radicals originate as well from LOX enzymes that catalyse dioxygenation of polyenoic fatty acids forming hydroperoxides. In the intestines, a substantial role is played by 5-LOXs, as it catalyses the oxidation of arachidonic acid. The hydroperoxides that are generated by LOX enzymes are then reduced by GPx [see review (Kuhn and Borchert 2002)].

Patients with CD, especially during an active phase of the disease, have higher plasma levels of lipid peroxidation products, as well as a decreased peroxidation potential and oxidative LDL level (Boehm et al. 2012). Although lipid peroxidation occurs in IBD patients, it may have different origin depending on the IBD type. Kruidenier et al. (2003) showed that in CD, lipid peroxidation is associated with mitochondrial superoxide dismutase (SOD) activity, suggesting the involvement of OH^•^ and O_2_^•−^, while the amount of lipid peroxidation products is associated with epithelial CAT expression and neutrophilic MPO activity in UC, suggesting a H_2_O_2_- and/or OCl^−^-mediated mechanism.

## Anti-oxidative mechanisms in GI tract

A non-harmful concentration of ROS/RNS is sustained by the anti-oxidative defence mechanisms, that include enzymes such as CAT, SOD or GPx and non-enzymatic endo- and exogenous scavengers like glutathione (GSH), transient ions (e.g. Fe^2+^, Cu^2+^) or flavonoids (Fig. 1). Noteworthy, it was demonstrated that the colonic enterocytes are characterised not only by higher ROS contents, as mentioned earlier, but also by higher concentration of CAT, SOD and GPx compared to small intestine tissue (Sanders et al. 2004).

Three mammalian SOD isoforms, copper/zinc (SOD1), mitochondrial (SOD2) and extracellular (SOD3), catalyse the reaction of O_2_^•−^ reduction to H_2_O_2_ (Fridovich 1997) [Fig. 1 reaction (7)]. SOD1 is a cyanide-sensitive homodimer localised mainly in the cytoplasm and to some extent in the nucleus, but absent in the mitochondria of epithelial cells and phagocytes (Pietarinen-Runtti et al. 2000; Kruidenier and Verspaget 2002). The mitochondria are protected from O_2_^•−^ by SOD2, which is vital for cell survival as mice lacking SOD2 gene die within several days after birth (Li et al. 1995). SOD3 dominates in plasma and interstitium (Kruidenier and Verspaget 2002) and has a high affinity to glycosaminoglycans like heparin (Marklund 1982).

Approximately 70 % of total SOD is expressed as SOD1, which not only dismutes O_2_^•−^, but can also convert H_2_O_2_ in the presence of copper ion, forming OH^•^ or peroxynitrate (Ischiropoulos and al-Mehdi AB 1995). SOD-produced H_2_O_2_ is converted to water in the reaction catalysed by CAT or GPx. CAT is widely expressed in the cytoplasm and peroxisomes of colonic epithelium and lamina propria and activated when concentrations of H_2_O_2_ increase, e.g. during inflammatory process. In contrast, H_2_O_2_ produced during normal cell metabolism is reduced by GPx in the presence of NADPH. GPx has higher affinity to H_2_O_2_ than CAT and also reduces lipid hydroperoxide levels, preventing peroxynitrite-mediated oxidation (Sies et al. 1997).

Currently, there are five isoforms of GPx, which belong to the group of selenium-dependent enzymes. GPx1 and GPx2 play an important role in the intracellular antioxidant defence, but in different layers of the gut; GPx1 is highly expressed in the colon lymphatic tissue and the lamina propria, submucosa, muscularis and serosa, but not the luminal epithelium, which is the area of the action of GPx2. GPx3 most likely contributes to the extracellular antioxidant defence of the intestinal mucosa, as it is secreted by intestinal epithelial cells (Esworthy et al. 1998; Tham et al. 1998). Recently, GPx4 has been detected in colonic and ileal tissues (Florian et al. 2010). This isoform is responsible for a repair of oxidatively damaged DNA by reducing thymine hydroperoxide and for scavenging phospholipid hydroperoxides and repressing lipid peroxidation (Bao et al. 1997; Seiler et al. 2008).

GPx enzymatic activity requires glutathione as a proton donor. GSH is a water-soluble tripeptide composed of the amino acids glutamine, cysteine and glycine, containing the cysteine-derived thiol group, which is a potent reducing agent. GSH is highly abundant in the cytoplasm (1–11 mM), nucleus (3–15 mM) and mitochondria (5–11 mM) and is the major soluble antioxidant in these cell compartments. GSH homeostasis in healthy tissues is sustained by de novo synthesis from cysteine, the regeneration of oxidised glutathione (GSSG), as well as from GSH uptake via sodium-dependent transport systems (Aw 2005). The reduction of two GSH particles in the presence of NADPH leads to the synthesis of GSSG. GSH is next regenerated from GSSG in the reaction mediated by GSH reductase (GRd) [Fig. 1 reactions (5) and (8)] or it is eliminated from the cell via export into the extracellular space (Bachhawat et al. 2013).

Several reports showed that the sufficient concentration of GSH in the jejunal and colonic epithelial cells prevents tissue degradation by eliminating harmful peroxides (Aw 2005), while the loss of GSH/GSSG redox balance contributes to tissue hyperplasia, mucosal inflammation and clinical symptoms of colitis (Tsunada et al. 2003). Oxidants like H_2_O_2_ were also shown to stimulate cysteine uptake and GSH synthesis (King et al. 2011). Furthermore, the promoter region of γ-glutamylcysteine synthetase, an enzyme involved in GSH synthesis, contains ROS-sensitive activator protein 1 (AP-1) binding site and an antioxidant response element (ARE) (Rahman et al. 1998). When activated, those regions increase GSH synthesis, thus enhancing anti-oxidative abilities of the cell (Aw 2005).

## Targeting oxidative stress in IBD

### Ulcerative colitis

ROS and NOS, as well as pro-inflammatory cytokines have a long-standing implication in both the aetiology and the progression of UC (Seril et al. 2003). A significant infiltration by neutrophils and increase in MPO levels was observed in the inflamed lamina propria of humans with UC in close approximation to the epithelia (Kruidenier et al. 2003). It was also shown in mice that the onset and severity of colitis were significantly attenuated by iNOS gene ablation (Krieglstein et al. 2001). In UC, iNOS is considered to be responsible for greatly increased production of NO in the epithelium and in foci of inflammation in association with nitrotyrosine (Singer et al. 1996). iNOS-derived NO stimulates TNF-α production in the middle and distal colon, which promotes the infiltration of neutrophils for example through stimulation of synthesis of intracellular adhesion molecule (ICAM) and P-selectin, therefore leading to colonic tissue damage (Yasukawa et al. 2012). Neutrophil recruitment and activation of key transcriptional signalling pathways like nuclear factor-kappa B (NF-kB) and AP-1 augment the inflammatory response and tissue damage (Brennan et al. 1995). When activated, NF-kB translocates to the nucleus, binds DNA and subsequently activates gene expression. The activated genes involved in mucosal inflammation include cytokines IL-6, IL-8 IL-1β, IL-10, TNF-α and ICAM (Yasukawa et al. 2012). Recently, Gan et al. (2005), documented an increased activation of NF-kB and high levels of the expression of interleukin IL-1β mRNA and IL-8 mRNA in human UC tissue.

Although UC is a well-known inflammatory bowel disease, the search for reliable disease markers continues. Studies reported higher concentration of serpin B1, a neutrophil elastase inhibitor which reduces H_2_O_2_-induced tissue damage in patients with inflamed UC (Uchiyama et al. 2012). Furthermore, those patients were more likely to possess a polymorphism in the CAT promoter region (C-262T) that alters CAT expression levels (Khodayari et al. 2013). Moreover, the proteomic characterization of inflamed colonic tissue demonstrated a relatively higher level of oxidative stress-response proteins like selenium binding protein, SOD and thioredoxin-dependant peroxide reductase, as well as higher expression of proteins implicated in energy generation like isocitrate dehydrogenase, l-lactate dehydrogenase B-chain, inorganic pyrophosphatase or enoyl-CoA hydratase, which could indicate inflammation-associated alterations in energy metabolism (Poulsen et al. 2012).

Clinical studies indicated that combined treatment of UC patients with oral mesalamine (2.4 g/day) plus N-acetyl-l-cysteine (0.8 g/day) for 4 weeks showed better clinical responses (66 vs 50 % in mesalamine alone group) accompanied by decreased levels of IL-8 and MCP-1 (Guijarro et al. 2008).

### Crohn’s disease

CD is characterised by reduced number of naive T cells and increased content of memory T cells, as well as higher expression of major histocompatibility complex (MHC) class II molecules in the colonocytes and in ileal epithelial cells (Ebert et al. 2005). At an early stage, patchy necrosis of the surface epithelium, focal accumulations of leukocytes adjacent to crypts and an increased number of intraepithelial macrophages and granulocytes are detected. Stimulated inflammatory cells produce ROS and RNS, but the mechanisms of free radical production and their sources in CD patients are complex. Previously, it was shown that blood polymorphonuclear neutrophils (PMNS) of patients with untreated CD have impaired infiltration ability, reduced SOD content, lower O_2_^•−^ production and therefore, decreased H_2_O_2_ generation (Verspaget et al. 1984; Verspaget et al. 1988; Curran et al. 1991). This is in line with Maor et al. (2008), who documented reduced release of O_2_^•−^ and lysozyme from neutrophils of patients with active but not stable CD. The authors speculated however, that the decreased superoxide anion production by the isolated PMNS might be caused by improper separation technique or the fact that the circulating substances present in serum exhausted their capacity for superoxide anion generation. Nevertheless, a positive correlation between the free radicals formation and pro-inflammatory cytokines content was described despite the fact that patients with active and stable CD had the anti-inflammatory medications in their clinical history (Maor et al. 2008). However, recent studies suggested that immune peripheral cells in patients with active CD have higher SOD activity and H_2_O_2_ production, increased lipid peroxidation, inhibited mitochondrial function and decreased CAT activity; interestingly those changes, apart from CAT activity, were reversed during disease remission showing an important role of mitochondria and oxidative stress in CD development (Beltran et al. 2010).

Also CD patients have higher ONOO^−^ content, a by-product of iNOS that is highly expressed in activated macrophages and neutrophils of colonic mucosa (Rachmilewitz et al. 1995).

The pathogenesis of CD may be as well associated with a decreased production of cytokines that suppress macrophage and T cell functions. For instance, intestinal tissue of CD patients is characterised by lower IL-4 mRNA expression, a cytokine, which delays O_2_^•−^ production in PMNS (Nielsen et al. 1996). Moreover, CD patients have lower content of anti-oxidative compounds, including tissue GSH, which participates in GPx-catalysed H_2_O_2_ reduction, as well as plasma ascorbic acid, α- and β-carotene, lycopene and β-cryptoxanthin (Miralles-Barrachina et al. 1999; Wendland et al. 2001; Maor et al. 2008). However, serum content of anti-oxidative enzymes like GPx seems to depend on the CD state; during CD remission, GPx activity is stable or lower, while its activity rises in active CD (Tuzun et al. 2002; Maor et al. 2008). The mouse models of UC and CD showed that an up-regulation of gene expression of GPx2 and down-regulation of aquaporin 8 (the facilitator of H_2_O_2_ diffusion) in the colon may play a protective role in defending against severe oxidative stress during IBD (Te Velde et al. 2008).

Apart from IL-4, several other cytokines play a role in CD, including TNF-α, IL-1β, IL-6 and IL-8 (Podolsky 2002). The release of cytokines is not only induced by ROS, but also by RNS. Recent study of (Rafa et al. 2013) showed an up-regulated NOS mRNA expression in peripheral blood mononuclear cells and colonic mucosa in patients with active CD and suggested a positive correlation between NOS-derived NO^•^ and IL-6, IL-17A and IL-23 plasma levels.

**Fig. 2:**
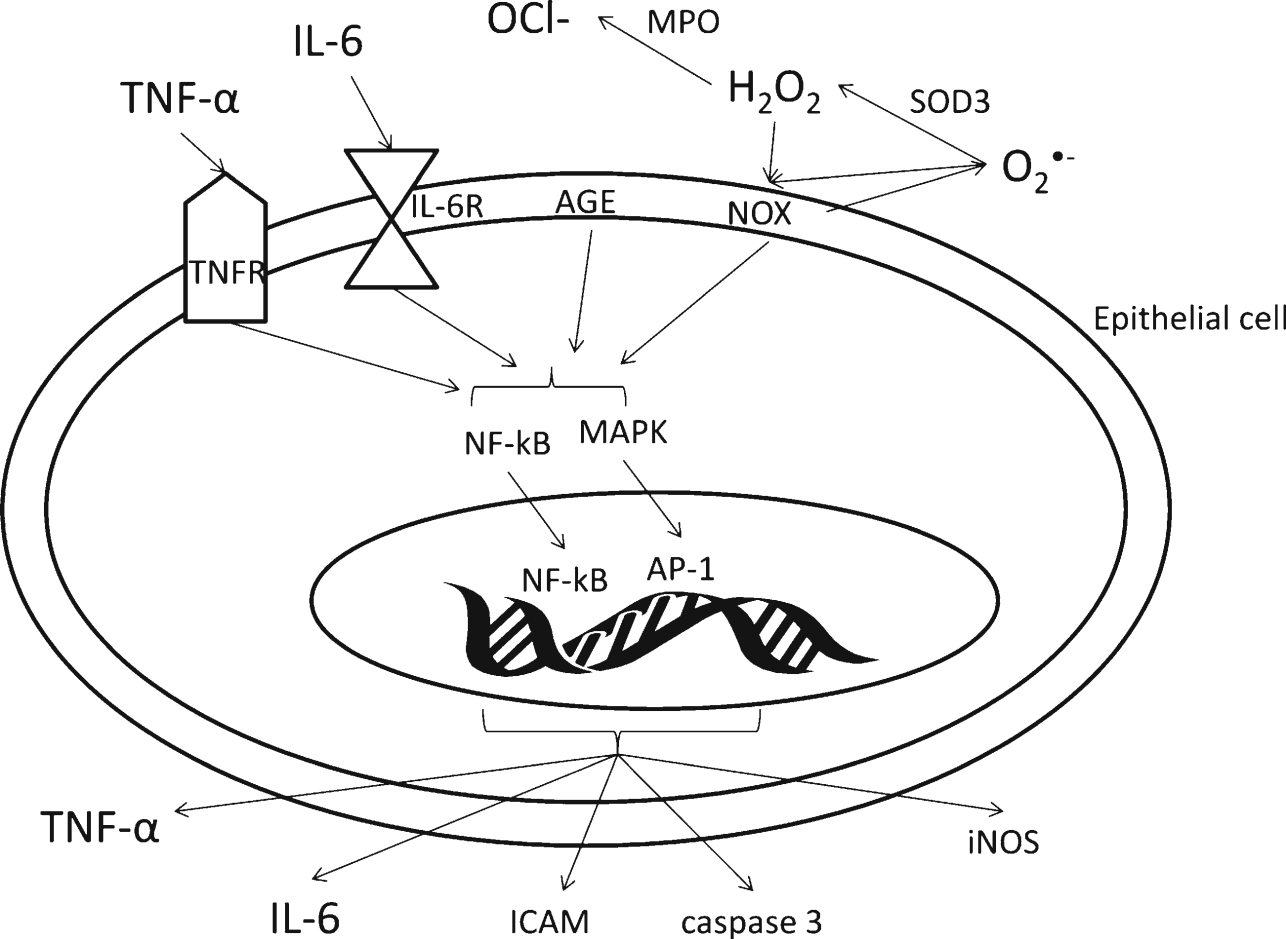
The influence of ROS and cytokines on signalling pathways in intestinal epithelial cells. *AGE* advanced glycation end products, *AP-1* activator protein 1, *ICAM* intracellular adhesion molecule, *IL-6* interleukin 6, *IL-6R* interleukin 6 receptor, *iNOS* inducible nitric oxide synthase, *NF-kB* nuclear factor-kappa B, *NOX* NADPH oxidase, *MAPK* mitogen-activated protein kinases, *OCl* ^*−*^ hypochlorite ion, *SOD3* extracellular superoxide dismutase, *TNF-α* tumour necrosis factor alpha, *TNFR* tumour necrosis factor receptor

The above-mentioned cytokines mediate their action via NF-kB and mitogen-activated protein kinase (MAPK) signalling pathways, and aberrant activation of NF-kB is involved in the pathogenesis of IBD (Schreiber et al. 1998). The participation of NF-kB and MAPK signalling pathways was presented in Fig. 2. Free radicals like superoxide anion are produced by NOX enzymes. The superoxide anion is converted to hydrogen peroxide by SOD3 and/or directly increases advanced glycation end products (AGE) content in plasma membrane of epithelial cells (Fig. 2). Both AGE and NOX, as well as pro-inflammatory cytokines e.g. IL-6 or TNF-α activate NF-kB signalling pathway leading to increased expression of caspase 3, ICAM, TNF-α or IL-6 genes, while activation of MAPK results in ameliorated AP-1 signalling molecule expression and increased production of iNOS, the uninhibited source of NO. Taken together, the inhibition of NF-kB or p38 MAPK may decrease cytokine production in CD and influence ROS/RNS production in CD patients, especially during the active phase of the disease (Waetzig et al. 2002).

### Colitis-associated colorectal cancer**—**ROS/RNS contribution

Carcinogenesis is generally a slow process and often takes decades from tumour initiation to diagnosis. The mutation and transformation process of a normal into a cancer cell can be triggered by accumulation of free radicals at the early stages and result in cancer progression. This might lead to an oxidative cellular damage or to an alteration in signalling pathways since ROS may act as signalling molecules.

Colorectal cancer remains the third most common cancer in both women and men worldwide (Chawla et al. 2013). It was demonstrated that during exogenous stress, the colon exhibits significantly greater oxidative DNA damage compared to the small intestine (Sanders et al. 2004). The oxidative environment results from excessive production of O_2_^•−^ in mitochondria, which can lead to the formation of other damaging agents like H_2_O_2_ and OH^•^. Moreover, it has been shown that mitochondrial respiration in the colon is less efficient than in other parts of intestines as the oxidation of butyrate, the primary energy substrate for colonocytes, yields 4.4 ATP/O_2_, while the oxidation of glutamine, the primary energy substrate for enterocytes, delivers 5.3 ATP/O_2_ (Wu et al. 1995).

Apart from influencing mitochondrial metabolism, ROS modifies cell cycle. It was indicated that in human colon adenocarcinoma cells ROS stimulate expression of p53, which—among other functions—plays a role of an oxidative response transcription factor, therefore causing S phase arrest (Sun et al. 2012).

The association between inflammation and cancer involves key inflammatory mediators, such as NF-kB-targeted gene products including TNFα, and COX-2. It was observed that down-regulation of COX-2 accelerated tissue healing in experimental colitis (Zwolinska-Wcislo et al. 2011) and the inhibition of COX-2 enzyme by therapeutic agents to prevent damage by ROS was thus proposed as a strategy for cancer chemoprevention. Other chemoprotective targets include the Kelch-like ECH-associated protein 1 (Keap1) and its binding protein, transcription factor NF-E2-related factor-2 (Nrf2), because of their role in regulating the antioxidant response element in response to oxidative stress (Chang et al. 2013). Nrf2 regulates the expression of anti-inflammatory enzymes like XO-1 and GSH transferase (Schuhmacher et al. 2011). Recently, it was indicated that Nrf2 deficiency in epithelial cells leads to oxidative stress and DNA lesions, accompanied by impairment of cell cycle progression, mainly G2/M-phase arrest (Reddy et al. 2008). This effect is decreased after addition of the redox status regulator, GSH, which is known to act as a growth regulator, whereas GSH deficiency results in growth arrest (Iwata et al. 1997). Additionally, Nrf2-mediated and ROS-dependant cell cycle arrest is accompanied by HO-1 expression, followed by p21 induction and prevention of neointimal hyperplasia (Kim et al. 2009).

Another strategy for cancer chemoprevention is to induce apoptosis via activation of MAPK pathways, in particular those involving c-Jun N-terminal kinase 1 (JNK) and p38 (Davis 2000; Ono and Han 2000). It was recently reported that mice with epithelial-deleted p38-MAPK in the colon had greater tumour development mediated by impaired cell cycle regulation (Wakeman et al. 2012). Also the GSH transferase, an enzyme incorporated in GSH metabolism, was shown to form protein-protein interactions with members of the MAPK pathway, thereby serving a regulatory role in the balance between cell survival and apoptosis (Scharlau et al. 2009).

The involvement of oxidative stress-regulated pathways in colon carcinoma was also confirmed in the in vitro experiments with free radical scavengers. For instance, the group of Hsu et al. (2007) showed that the administration of N-acetylocysteine (NAC), a ROS scavenger, reduced colonic cancer cell apoptosis via inhibition of JNK, p38 MAPK and activation of c-jun. Also, a pharmacological inhibition of ERK and p38 MAPK may decrease HO-1 up-regulation in colonic cells (Park et al. 2010). The induction of HO-1 gene expression is an important event in cellular response to pro-oxidative and pro-inflammatory compounds. However, further studies are necessary to determine the role of oxidative stress and oxidative stress-stimulated signalling pathways in colitis-associated colorectal cancer.

## Clinical view of the anti-oxidative role of drugs used in IBD treatment and their influence on IBD outcome

Current treatment strategies for moderate-severe IBD consist immunosuppressants, corticosteroids and anti-TNF-α antibodies. Therapeutic effect of those drugs is in part contributed to their anti-inflammatory and anti-oxidative properties. Immunosuppressants and corticosteroids possess direct free radical-scavenging abilities while anti-TNF-α antibodies decrease TNF-α concentration having indirect anti-oxidative effect.

### Sulfasalazine and mesalazine

Sulfasalazine is a potent cysteine transporter inhibitor composed of 5-aminosalicylic acid and sulfapyridine that has been routinely used in the clinical therapy of IBD (Gan et al. 2005). After oral intake, sulfasalazine is split by intestinal flora into sulfapyridine and mesalazine (Rijk et al. 1988). Like salicylates, the anti-inflammatory potential of sulfasalazine may be reflected by its influence on the release of adenosine, which controls oxidative potential, and by the effect of sulfasalazine on pro-inflammatory compounds content and free radicals generation. It was indicated that in clinical studies, a 6-week treatment with sulfasalazine to patients with mildly and moderately active UC resulted in a significant decrease of gut inflammation (Chen et al. 2005). This effect can be explained by sulfasalazine influence on ROS and pro-inflammatory cytokines content. It was shown that sulfasalazine decreased ROS concentration (Guo et al. 2011). In patients with moderate UC treated with sulfasalazine (2–4 g/day) for 8–36 weeks, a down-regulated activity of NF-kB accompanied by decreased expression of pro-inflammatory IL-1β mRNA and IL-8 mRNA was observed (Gan et al. 2005). When stimulated, NF-kB signalling pathway activates genes for e.g. pro-inflammatory cytokines production. Therefore, the down-regulation of NF-kB activity under sulfasalazine treatment is desired. Interestingly, in patients with moderate UC, decreased concentration of NF-kB was independent of IkBα level, which is a regulatory protein that inhibits NF-kB by trapping it in the cytoplasm (Gan et al. 2005). In response to a stimulus, IkBα degrades and rapidly returns to the original level, what traps NF-kB and keeps it inactive, therefore indirectly inhibiting NF-kB effects (Scherer et al. 1995).

Deactivation of NF-kB by sulfasalazine was also described in the in vitro models. For instance, sulfasalazine-mediated inhibition of NF-kB induced apoptosis of T lymphocytes (Liptay et al. 1999) and macrophages (Brindley et al. 1996). In the macrophages, sulfasalazine also stimulates phospholipase D, an enzyme involved in the regulation of cell signalling and oxidant stress, and the generation of phosphatidate (Brindley et al. 1996).

Sulfasalazine was also shown to inhibit extracellular release of pro-inflammatory secretory phospholipase A2 (Pruzanski et al. 1997).

Mesalazine (5-ASA), a metabolite of sulfasalazine, is widely used for the treatment of UC. At the cellular level 5-ASA reduces oxidative stress by inhibiting O_2_^•−^ and H_2_O_2_ production, as well as preventing mucosal GAPDH inhibition (Kimura et al. 1998; Campregher et al. 2010). Clinical trials indicated that in patients with UC, 4-week treatment with 5-ASA (2.4 g/day) plus N-acetyl-l-cysteine (0.8 g/day) not only improved clinical response but also correlated with decreased blood TNF-α, IL-6 and IL-8 concentration, as well as improved GSH content (Guijarro et al. 2008). 5-ASA administrated alone also improved clinical outcome, but with little effect on IL-6 and IL-8 content and with no influence on GSH and TNF-α concentration (Guijarro et al. 2008). Also in patients after ileocolonic resection of CD, a 6-month 5-ASA (6 g/day) prevented the CD recurrence, but it did not reduce pro-inflammatory cytokine content; the concentration of mucosal TNF-α, IL-1β and IL-6 was increased (Yamamoto et al. 2009).

The possible difference in action between sulfasalazine and mesalazine in patients with IBD was recently described in a retrospective cohort study (Masuda et al. 2012). The authors observed that mesalazine group (*n* = 303, 250–40,00 mg/day for 111 days) had greater haematological adverse effects, expressed by lower white blood cells and platelet counts and higher mean serum urea nitrogen level than the sulfasalazine group (*n* = 67, 250–6,000 mg/day for 116 days). Therefore, we may speculate that the haematological changes after 5-ASA therapy may influence free radical generation and pro-inflammatory cytokine content in IBD patients.

It should be also noticed that 5-ASA potently inhibits peroxynitrite-mediated DNA strand breakage, scavenges peroxynitrite and affects peroxynitrite-mediated radical formation responsible in part for 5-ASA anti-inflammatory and anti-cancer effects (Graham et al. 2013).

As discussed above, it may be suggested that sulfasalazine seems to be more effective than mesalazine. However, additional studies are necessary to evaluate the efficacy of sulfasalazine and 5-ASA in oxidative stress.

### Corticosteroids

Systemic corticosteroids are highly effective at inducing clinical remission of UC and CD. Currently, a second generation of corticosteroids, which includes budesonide, prednisone or beclomethasone dipropionate, is in clinical use and they seem to possess fewer side effects in patients treated for UC and CD. Studies indicated that glucocorticoid therapy effectively inhibited neutrophil activity, reflected by decreased MPO and neutrophil elastase serum contents in paediatric IBD (Makitalo et al. 2012).

The anti-oxidative and anti-inflammatory action of glucocorticoids can be explained by their influence on NF-kB. It was demonstrated that glucocorticosteroids (e.g. prednisolone 0.75 mg/kg/day for 3 weeks) strongly inhibit intestinal NF-kB activation by stabilising the cytosolic IkBα activation in tissue from patients with colitis (Ardite et al. 1998; Schreiber et al. 1998). Although helpful in decreasing ROS, corticosteroids do not seem to reduce the mucosal expression of NOS in patients with UC (Leonard et al. 1998). However, a recent study demonstrated a significant inhibition of NOS mucosal level and rectal NO production in patients with UC (*n* = 22) and CD (*n* = 24) treated with prednisolone (0.5–1 mg/kg orally for 1 month) (Ljung et al. 2006). Therefore, the effect of glucocorticosteroids on NOS and NO synthesis has to be further analysed.

It should also be stressed that corticosteroids may have a different effect on signalling pathways activity in CD patients who are steroid sensitive or steroid insensitive. Glucocorticoid treatment to steroid-sensitive patients lead to an activation of NF-kB, AP-1 and p38 MAPK mainly in lamina propria macrophages, while glucocorticoids mediated those changes mostly in epithelial cells in steroid-resistant patients (Bantel et al. 2002). Thus, steroid resistance is associated with increased epithelial activation of the above-mentioned pathways that may inhibit the anti-inflammatory glucocorticoid-induced action and accelerate disease progress.

### Cyclosporine

Cyclosporine A, a calcineurine inhibitor, is an immunosuppressive drug which was shown to suppress the production of IL-2 and IL-3, inhibit chemotaxis of neutrophils and induce apoptosis in T cells of patients with UC (Ina et al. 2002; Kountouras et al. 2004). Cyclosporine A also decreased the number of neutrophils and mononuclear cells in colonic tissue and inhibited cytotoxic activity of T cells and mucosal chemokine activity in humans (Ina et al. 2002). When administered to humans, cyclosporine A binds to cyclophilin A, whose gene expression was shown to be up-regulated in the crypt epithelia of UC patients (Kim et al. 2006). The cyclosporine-cyclophilin A complex decreases TNF-α and IL-6 concentration by inhibiting the activity of NF-kB and MAPK signalling pathways in monocytes, therefore altering inflammatory processes (Yuan et al. 2010). However, no association was found between clinical response and whole blood cyclosporine A concentration in patients receiving both high (>5 mg/kg/day) and low (<5 mg/kg/day) oral cyclosporine A dose (Egan et al. 1998).

### Anti-TNF-α antibodies

Infliximab is a monoclonal antibody against serum and membrane-bound TNF-α, which decreases TNF-α concentration in colonic mucosa in patients with UC (Hart and Ng 2010). Infliximab treatment has been shown to decrease inflammation which improved mucosal healing in patients with UC via healing of the goblet cells and reducing abnormal mucus formation and secretion, which finally led to the recovery of the villi components (Fratila and Craciun 2010). Studies on 32 patients suffering from UC for about 4 years and treated with infliximab in repeated intravenous infusions at 0, 2 and 6 weeks expressed lower mRNA of TNF-α and INF-γ (Olsen et al. 2009). Moreover, UC remission was observed in eight patients after infliximab treatment. The colon tissue of UC remission patients was characterised by lower number of macrophages and lymphocytes; however, the level of TNF-α positive cells was unchanged (Olsen et al. 2009). Those changes can be attributed to inhibition of TNF-α generation and modulation of TNF-α stimulated signalling pathway. Apart from reducing mRNA for TNF-α, infliximab decreased T lymphocyte and macrophage content and down-regulated the expression of IFN-γ without affecting IL-10 and IL-4 mRNA (Olsen et al. 2009). Infliximab introduced to patients at a dose of 5 mg/day for 2–4 weeks inhibited neutrophil activity, reflected by lower neutrophil elastase level, but not as efficiently as glucocorticoids at a dose of 0.8 mg/kg/day (Makitalo et al. 2012). Moreover, infliximab therapy decreased the up-regulation of leukocyte cell adhesion molecules and the inflammatory cell number in colonic lamina propria (Arijs et al. 2011). Furthermore, semi-chronic administration of anti-TNF-α antibodies increased blood contents of regulatory T cells and their suppressive function (Boschetti et al. 2011).

Unlike UC, CD is characterised by increased mucosal concentrations of TNF-α even during disease remission (Raddatz et al. 2005). Infliximab treatment lead to lower global numbers of CD4+ and CD8+ T lymphocytes and CD68, a marker of monocytes/macrophages (Baert et al. 1999). It also decreased mucosal expression of T regulatory cells, counted as forked box P3 (FoxP3) level (Li et al. 2010). Therefore, targeting TNF-α generation in CD patients seems to be crucial. It was presented that infliximab treatment (5 mg/kg, every 8 weeks for 6 months) to patients after resection of CD showed a decrease in mucosal IL-1β, IL-6 and TNF-α which contributed to the suppressive effect on clinical and endoscopic disease activity (Yamamoto et al. 2009). Similarly, six injections of adalimumab to 70 CD patients (80 mg at week 0 and then 40 mg every 2 weeks as subcutaneous injections), another anti-TNF-α antibody, for 10 weeks to patients with CD significantly decreased mucosal mRNA level of TNF-α, INF-γ, IL-17A and IL-23 (Rismo et al. 2012). The decreased level of IFN-γ may result from its reduced secretion by T cells and depletion of TNF levels (Agnholt and Kaltoft 2001). Decreased cytokines concentration can directly influence ROS/RNS production by inflammatory cells or indirectly modulate ROS-stimulated signalling pathways activity. However, further studies indicating the role of anti-TNF-α antibodies drugs on ROS/RNS production in IBD are necessary.

### Thiopurines

The thiopurines, which include azathioprine (AZA) and mercaptopurine (MP), remain a mainstay in the management of IBD. Thiopurines are relatively efficacious—nearly 70 % of patients with steroid-dependent IBD achieve and maintain remission (Pearson et al. 1995). However, their use is limited because of their high intolerance level and the risk of adverse reaction, which is between 15 and 28 % (D'Haens et al. 1999). When metabolised, AZA is converted to 6-thioguanine nucleotides (6-TG), which is incorporated into cellular DNA and may be accumulated therein. It was shown that IBD patients have detectable 6-TG DNA in lymphocytes (Cuffari et al. 1996). It was recently described that 6-TG DNA, produced in patients under AZA treatment, increases DNA susceptibility to ROS produced in a biological context (Daehn and Karran 2009). Moreover, in the same study, the authors demonstrated that macrophages which contain DNA 6-TG are at risk from self-inflicted DNA 6-TG oxidation and their sustained high level of endogenous ROS swiftly leads to cell death.

Nevertheless, AZA remains one of the most efficient anti-inflammatory drugs that decreases infiltration of inflammatory cells into the ileal mucosa in CD patients and facilitates mucosal healing (D'Haens et al. 1999).

## Future therapies based on anti-oxidative and anti-inflammatory drugs**—**brief review of experimental data

The severity of colitis can be modified therapeutically by drugs that influence free radicals generation, neutrophil infiltration and pro-inflammatory agents’ production. Uraz et al. (2013) showed that oral administration of NADPH oxidase inhibitor, NAC, to mice with acetic acid-caused UC significantly decreased pro-inflammatory cytokine concentration and lipid peroxidation, as well as elevated GSH and SOD content (Table 2). Similar results were obtained in rat model of acetic acid-induced colonic inflammation (Nosal'ova et al. 2000; Cetinkaya et al. 2005). Moreover, NAC amplified protective effect of a well-established anti-inflammatory agent, 5-ASA, used in UC patients, and decreased COX-2 gene expression and prostaglandin E2 level, therefore influencing colon nitrate generation and iNOS activity (Ancha et al. 2009). NAC alone reduced iNOS level in ulcerative distal colon (Seril et al. 2002).

**Table 2:** Anti-oxidative and anti-inflammatory effects of therapeutics used in ulcerative colitis treatment

Antioxidants and anti-inflammatory drugs in the treatment of IBD	Role	Reaction No. in Fig. 1	Reference
	Pre-clinical studies		
N-acetylocysteine	↓MPO, ↑GSH in colon lesions ↓iNOS in distal colon lesions ↓MPO, ↑GSH, SOD, ↔ CAT in colon lesions ↓COX-2, PGE2, nitrate concentration ↓lipid peroxidation, ↑GSH, SOD in ulcerative colitis ↓COX-2 and iNOS mRNA in colon lesions ↓iNOS activity in UC ↑GSH/GSSG ratio in intestinal subepithelial myofibroblasts in CD	(5)	(Nosal'ova et al. 2000) (Seril et al. 2002), (Cetinkaya et al. 2005) (Ancha et al. 2009) (Uraz et al. 2013), (Nosal'ova et al. 2000), (Cetinkaya et al. 2005) (Ancha et al. 2009) (Seril et al. 2002), (Romagnoli et al. 2012)
Lipoic acid	↑GSH, ↓MPO and lipid peroxidation in ileum and colon	(5)	(Kolgazi et al. 2007)
Curcumin and ellagic acid	↓MPO, COX-1, COX-2, LOX, TNF-α, IFN-γ, iNOS tissue level in CD		(Baliga et al. 2012), (Rosillo et al. 2011)
Tetradecylthioacetic acid	↓iNOS, TNF-α and IL-6 mRNA in ulcerative colitis		(Bjorndal et al. 2013)
Tributyrin	↑TGF-β and IL-10 in lamina propria		(Leonel et al. 2012)
Lactulose, a molecular hydrogen inducer	↓TNF-α, IL-1β, MPO in colon lesions ↓ONOO-, OH• in colonic lesions	(3)	(Chen et al. 2013) (Ohsawa et al. 2007)
Ectoine	↓IL-1α, IL-6, IL-8 and TNF-α		(Sydlik et al. 2009; Abdel-Aziz et al. 2013)
	Clinical studies		
Mesalazine	↓O2•^−^, H2O2 in UC ↓IL-6, Il-8, ↔GSH, TNF-α in UC ↑TNF-α, IL-1β and IL-6 in mucus of CD	(7)	(Campregher et al. 2010) (Guijarro et al. 2008) (Yamamoto et al. 2009)
Sulfasalazine	↓ROS, ↓IL-1β and IL-8 mRNA		(Guo et al. 2011) (Gan et al. 2005)
Glucocorticoids	↓MPO and neutrophil elastase in paediatric IBD		(Makitalo et al. 2012)
Cyclosporine	↓IL-2, IL-3		(Kountouras et al. 2004)
Infliximab	↓TNF-α in colonic mucosa ↓INF-γ mRNA in inflammatory cells in colitis		(Fratila and Craciun 2010) (Olsen et al. 2009)
Adalimumab	↓TNF-α, INF-γ, IL-17A, IL-23 mRNA in colonic mucosa of CD patients		(Rismo et al. 2012)

*CAT* catalase, *CD* Crohn’s disease, *GRd* glutathione reductase, *GSH* reduced glutathione, *GSSG* oxidised glutathione, *GPx* glutathione peroxidase, *H*_*2*_ *O*_*2*_ hydrogen peroxide, *IBD* inflammatory bowel disease, *IL* interleukin, *IFN-γ* interferon gamma, *LOX* lipooxygenase, *MPO* mieloperoxidase, *NO* ^*•*^ nitric oxide, *iNOS* inducible nitric oxide synthase, *NOX* NADPH oxidase, *ONOO* ^*−*^ peroxynitrate, *O*_*2*_^*•−*^ superoxide anion, *OH* ^*•*^ hydroxyl radical, *PGE*_*2*_ prostaglandin E_2_, *SOD1* copper/zinc superoxide dismutase, *SOD2* mitochondrial superoxide dismutase, *SOD3* extracellular superoxide dismutase, *TNF-α* tumour necrosis factor alpha, *UC* ulcerative colitis, *XO* xanthine oxidase

Romagnoli et al. (2012) reported that NAC prevents TNF-α-induced GSH/GSSG ratio depletion in intestinal subepithelial myofibroblasts isolated from patients with active CD. The improvement of cell redox status negatively correlated with secreted matrix metalloproteinase-2, a compound responsible for a dysfunction of epithelial barrier in CD patients.

Recently, another natural anti-oxidant, lipoic acid, was shown to decrease tissue lipid peroxidation, MPO activity and increase GSH content in rats with ileitis or colitis (Kolgazi et al. 2007). Similarly, curcumin, an active ingredient of an Indian spice, and ellagic acid, a natural polyphenol, were used in IBD treatment for their scavenging activity to free radicals, inhibition of MPO, COX-1, COX-2, LOX, TNF-α, IFN-γ, iNOS and positive influence on multiple signalling pathways, especially the MAPK and NF-kB [see review of Rosillo et al. (2011) and Baliga et al. (2012)].

Bjorndal et al. (2013) observed that fatty acid analogue tetradecylthioacetic acid, an anti-inflammatory and antioxidant agent, reduced colonic oxidative damage by decreasing iNOS, TNF-α and IL-6 at mRNA level. Other therapeutics like tributyrin reduced mucosal damage and neutrophil and eosinophil mucosal infiltration, which was associated with a higher percentage of regulatory T cells and higher levels of TGF-β and IL-10 in the lamina propria (Leonel et al. 2012). Inhibition of TNF-α and IL-1β during experimentally induced colitis can also be observed after oral administration of molecular hydrogen (H_2_) inducers, like lactulose (Chen et al. 2013). The protective role of molecular hydrogen against oxidative stress is associated with H_2_ ability to neutralise the ONOO^−^ and OH^•^ (Ohsawa et al. 2007).

A natural compound ectoine found in several species of bacteria inhibits colitis by blocking nuclear translocation of NF-kB and MAPK and down-regulation of the expression of the pro-inflammatory cytokines like IL-1α, IL-6, IL-8 and TNF-α (Sydlik et al. 2009; Abdel-Aziz et al. 2013). Similar results were documented for parthenolide, an herbal compound, which reduced the production of TNF-α and IL-1β via influencing phosphorylation and subsequent degradation of NF-kB inhibitory protein IkBα in mice (Zhao et al. 2012). As NF-kB is an oxidative stress-activated pathway, its inhibition may decrease ROS production. The activity of NF-kB pathway may also be influenced by compounds that constitute an energy source for colonic epithelial cells, like butyrate. It was indicated that in colonic epithelial cells and mucosal biopsies of CD patients, butyrate lowered LPS-induced ROS concentration and down-regulated gene expression and protein content of NF-kB, TNF-α, COX-2 and ICAM-1 (Russo et al. 2012). In addition, the inhibition of NF-kB activation affects cell apoptosis by silencing of mRNA expressions of Fas/FasL, Bax and caspase-3, and activated Bcl-2 genes in intestinal epithelial cells (Liu and Wang 2011). The inhibition of apoptosis prevents excessive loss of epithelial cells and therefore, intestinal injury.

Treatment of UC can also target promoter regions for chemoprotective genes, like heme oxygenase-1 (HO-1). Recently, Yukitake et al. (2011) reported that activation of ARE-mediated gene expression with BTZO-15 reduced the ulcerated area by increasing expression of HO-1, suppressing NO-induced cell death and ameliorating rectal metalloproteinase activity. BTZO-15 is a derivative of BTZO-1 (BTZO-1, 2-pyridin-2-yl-4H-1,3-benzothiazin-4-one) that possesses cytoprotective effect by elective bounding to macrophage migration inhibitory factor (MIF), and increasing in GSH transferase mRNA expression (Kimura et al. 2010).

Recently, Biagioni et al. (2006) reported that defective neutrophil function in patients with CD can be restored by granulocyte-macrophage colony-stimulating factor (GM-CSF), which activates respiratory burst and improves cell viability. GM-CSF is necessary for proper mucosal barrier function, and patients with elevated GM-CSF antibody exhibit an increase in bowel permeability and disease severity vs. patients with CD with lower levels of GM-CSF antibody (Nylund et al. 2011).

## Conclusion

The results of both clinical and experimental studies suggest a potential involvement of ROS and RNS in the pathomechanism of IBD and colitis-associated colorectal cancer. However, it remains unclear whether the increased oxidative stress in the gut environment results from failing metabolism, especially in mitochondria or is a reason of decreased local scavenging capacity. Also, an excessive activation of macrophage, PMN infiltration and/or activation can contribute or cause local increase in free radicals production. Probably all above-mentioned mechanisms of excessive free radicals production are engaged in the aetiology and/or exacerbation of IBD and colitis-associated colorectal cancer. Therefore, further analyses are necessary to accumulate a larger amount of data on the anti-oxidative and anti-inflammatory role of currently used therapeutics and their interference with ROS/RNS-stimulated signalling pathways.

Clinical studies with existing and potential anti-IBD drugs, especially those employing natural antioxidants show promising outcomes for IBD and colorectal cancer treatment (Rosillo et al. 2011; Baliga et al. 2012). However, since numerous intestinal factors like bacteria, digestive enzymes or food metabolites can change the anti-oxidative properties of drugs potentially inactive, further clinical trials are necessary. The knowledge on highly specific anti-oxidative effects of currently used therapeutics and novel agents may provide significant clinical benefits in the treatment and relapse of IBD.

It is likely that, in the near future, combination of currently used pharmaceutics with natural and synthetic potent anti-oxidative compounds, like lipoic acid or curcumine, will become a strategy of choice in IBD treatment.

## List of non-standard abbreviations

AGE: Advanced glycation end products
AP-1: Activator protein 1
ARE: Antioxidant response element
CAT: Catalase
CD: Crohn’s disease
eNOS: Endothelial nitric oxide synthase
GI: Gastrointestinal
GRd: Glutathione reductase
GSH: Reduced glutathione
GSSG: Oxidised glutathione
GPx: Glutathione peroxidase
H_2_O_2_: Hydrogen peroxide
HO: Heme oxygenase
IBD: Inflammatory bowel diseases
ICAM: Intracellular adhesion molecule
IL: Interleukin
IkB: Alpha- inhibitor of kB alpha of NF-kB
IFN-γ: Interferon gamma
iNOS: Inducible nitric oxide synthase
LOX: Lipooxygenase
MAPK: Mitogen-activated protein kinases
MPO: Myeloperoxidase
NF-kB: Nuclear factor-kappa B
NOS: Nitric oxide synthase
NOX: NADPH oxidase
NO^•^: Nitric oxide
ONOO^−^: Peroxynitrate
O_2_^•^: Superoxide anion
OCl^−^: Hypochlorite ion
OH^•^: Hydroxyl radical
PMNS: Polymorphonuclear neutrophils
ROS: Reactive oxygen species
RNS: Reactive nitrogen species
SOD1: Copper/zinc superoxide dismutase
SOD2: Mitochondrial superoxide dismutase
SOD3: Extracellular superoxide dismutase
UC: Ulcerative colitis
TNF-α: Tumour necrosis factor alpha
TNFR: Tumour necrosis factor receptor
XO: Xanthine oxidase
